# Elasticity meets topology

**DOI:** 10.1093/nsr/nwae131

**Published:** 2024-04-04

**Authors:** Guancong Ma

**Affiliations:** Department of Physics, Hong Kong Baptist University, China

## Abstract

Harnessing the unique vectorial properties of elastic waves, Wu *et al.* find new degrees of freedom for realizing novel topological phases.

We are surrounded by environments and items that are made from solid materials in our daily life. Any physical contact with any items is a mechanical disturbance that produces mechanical vibrations. This underlines the crucial role that such vibrations play in our daily interactions, highlighting the need for a deeper understanding and improved manipulation of these fundamental phenomena. The effective control and manipulation of these mechanical vibrations are, therefore, a topic of paramount importance.

Mechanical vibrations propagate as elastic waves. Unlike acoustic or electromagnetic waves, elastic waves in solid materials can be transverse or longitudinal in character. What is more, the wave modes are oftentimes a combination of both. For example, elastic waves on a thin plate can be in-plane compressional modes (dominantly longitudinal), in-plane shear modes or flexural modes that manifest as bending motions, as shown in Fig. 1(a–c) [1]. Such characteristics make elastic waves potentially fertile ground for wave physics but, in the meantime, this also means that elastic waves are inherently difficult to manipulate.

In recent years, the study of elastic waves has been rejuvenated by the concept of topological matters [2,3]. However, owing to the complexity of elastic waves, the potential topological concepts remain under-explored in the realm of elastic waves. In a recent work, Wu *et al.* explored the richness of elastic waves and successfully realized a topological elastic-wave crystal [4]. They first designed a plate-type elastic-wave crystal. Each unit cell is a thin plate decorated with a stub, which is essentially a short rod. Mode analysis found that the flexural mode and the longitudinal mode are coupled and form a quadratic degenerate point. Then, by joining two identical crystals together to form a double-layer structure, all three (flexural, shear, longitudinal) modes are engaged to form 4-fold degenerate quadratic point. Remarkably, they found that the 4-fold degeneracy can be lifted by using a chiral inter-plate coupling design and doing so gives rise to a topological bandgap. In other words, the topological bandgap is a consequence of the rich vectorial nature of the elastic modes. When the double-layer lattice is truncated, two topological states appear at the boundary. Intriguingly, these topological states are each associated with a rotational mode of the stub. The rotational modes are clockwise and anticlockwise in chirality, resembling the circular polarization, or spin, of light. The rotational modes, as shown in Fig. 1(d, e), play the role of pseudospins—two orthogonal degrees of freedom that enable chiral transport of the topological edge states.

**Figure 1.**
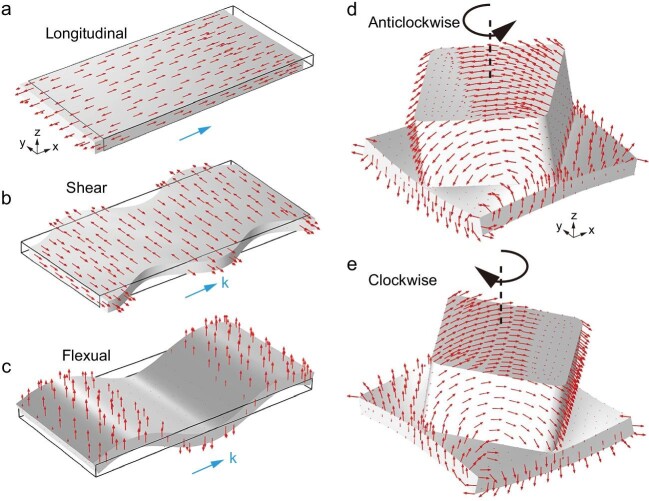
(a–c) The three types of elastic waves on a thin plate. The (a) longitudinal and (b) shear modes are dominantly in-plane vibrations, whereas the flexural mode is characterized by the bending motion (c). (d and e) The two counter-rotating modes play the role of pseudospins that enable the topological properties and helical transport reported in Ref. [4].

The authors additionally performed investigations into how different boundary conditions affect the topological states. Their experiments show that the chiral topological states not only feature backscattering immunity, but also exist for both free and clamped boundary conditions. Such outstanding robustness means that the topological concept is exceptionally suitable for applications that need to cope with complicated situations such as perturbations from the environment.

The work by Wu *et al.* is a successful crossover between the novel concepts from topological physics and the rich complexity inherent in the elasticity of solid materials. Their findings suggest that harnessing this complexity could provide unparalleled control over the propagation of elastic waves. This work has the potential to open new doors for a broad range of scenarios ranging from nanophononics to mesoscopic mechanics to robotics, civil engineering and seismic engineering.
